# Predictive Value of Health Literacy on Health‐Promoting and Protective Behaviours in Turkish Nurses and Allied Health Workers

**DOI:** 10.1002/nop2.70549

**Published:** 2026-05-25

**Authors:** Dilan Deniz Akan, Akgün Yeşiltepe

**Affiliations:** ^1^ Department of Internal Medicine Nursing, Faculty of Health Sciences Manisa Celal Bayar University Yunusemre Manisa Türkiye; ^2^ Munzur University Faculty of Health Science Tunceli Türkiye

**Keywords:** health literacy, healthcare workers, health‐promoting behaviours, nurses, protective behaviours

## Abstract

**Aim:**

This study aims to investigate the extent to which Turkish nurses and allied health workers' levels of health literacy predict health‐promoting and protective behaviours.

**Desing:**

A descriptive and cross‐sectional study design was used.

**Methods:**

The study was conducted with 403 Turkish nurses and allied health workers working in different regions of Türkiye between January and May 2024. JASP 0.18.3.0, Jamovi 2.3.21, and SPSS 26 and AMOS26 programs were used for data analysis. Descriptive statistics and simple and multiple regression analysis were used in the analysis of the data.

**Results:**

A positive, moderately significant relationship was found between health literacy and health‐promoting and protective behaviours (*r* = 0.460, *p* < 0.001). Regression analysis results showed that health literacy explained 21.1% (*R* ^2^ = 21.1) of the change in health‐promoting and protective behaviours and was a significant predictor of health‐promoting and protective behaviours.

**Patient or Public Contribution:**

Understanding the predictive effect of health literacy and preventive health behaviours can guide practices and policies that will improve the level of health literacy and preventive health behaviours in healthcare workers. Improving their health literacy and preventive health behaviours can help patients and the general public increase their health literacy levels and gain positive health behaviours.

## 1 Introduction

Health literacy (HL), which first entered the literature in 1974 with Simond's article, is defined as ‘the capacity to access the information necessary for individuals to make appropriate decisions about health in order to promote and maintain good health; to understand, interpret and apply this information in their daily lives’ (Batterham et al. 2016; Dogan and Çetinkaya 2019). HL is closely related to the individual and the society and is accepted as a social determinant of health (Durmaz et al. 2016; Budhathoki et al. 2019).

Studies in the literature show that the desired levels of HL have not been reached in the world and in our country (Suka et al. 2015; Tavousi et al. 2016; Schaeffer et al. 2017; Rajah et al. 2019). In the HL survey covering eight countries in Europe, it was determined that approximately half of the participants had inadequate or problematic HL levels (Sørensen et al. 2015). In the United States of America, it was reported that 88% of adults had limitations in HL (Loan et al. 2018). In the HL study conducted in Türkiye, 68.9% of the participants were reported to have inadequate or problematic limited HL (Republic of Turkey Ministry of Health General Directorate of Health Promotion 2018). Since low HL level prevents achievement of desired health outcomes, it is recognized as an important public health problem all over the world (Zhang et al. 2022).

Low HL level affects all segments of the society negatively. Low HL level contributes to poor health, poor drug compliance, poor disease management, repeated hospitalisations, increased inequality in health and negative use of health care resources (Tsai et al. 2018; Liu et al. 2020). HL has critical importance in individuals' access to health‐related information, effective use of this information, protection of health and adoption of health‐promoting behaviours (Ho et al. 2017; Kazak et al. 2021). When the level of HL of the individual and the society increases, informed decisions are made and health services are used more effectively. Health‐promoting and protective behaviours increase (Ayaz‐Alkaya et al. 2020; Kinoshita et al. 2024).

Health‐promoting and protective behaviours (HPPB), which are defined as the power of individuals to avoid negative health behaviours and increase their control over their own health, are an important strategy for maintaining health and preventing chronic diseases (Degerli and Yigit 2020). HPPB are effective in achieving a better level of health and increasing the level of welfare (Choi et al. 2024).

Healthcare workers act as role models for the society and individuals to whom they provide healthcare services. Studies have shown that healthcare workers' own health practices have significant effects on the community and the individuals they serve (Iriarte‐Roteta et al. 2020; Wilandika et al. 2023). From this point of view, healthcare workers may assume a key role in increasing the level of HL in the community and developing HPPB (Soykan and Sengul 2021). However, research in the literature indicates that nurses and healthcare workers' knowledge and awareness of HL are not yet sufficient (Lewis et al. 2014; Rajah et al. 2017; Nantsupawat et al. 2020). In one study, it was reported that only 55.3% of nurses knew the concept of HL, and 51.7% evaluated their HL knowledge level as “moderate” (Nantsupawat et al. 2020). Furthermore, research emphasizes the need for nurses and other healthcare workers to have a better understanding of HL (Ozen et al. 2019).

When the studies in the literature are examined, it is seen that the studies examining HL and HPPB in Turkish nurses and allied health workers are quite limited in the literature (Soykan and Sengul 2021) and the studies conducted are generally focused on nurses (Zeng et al. 2021; Yogurtcu and Ozturk Haney 2022). However, health is a teamwork. The role of all members of the team is very important. Moreover, it is seen in the literature that the effect of HL on HPPB is not sufficiently understood in the context of Turkish nurses and allied health workers. In the context of Turkish nurses and allied health workers, a clear understanding of the effect of HL on health behaviours can play an important role in increasing the level of HL and achieving health promotion goals. In this way, it can contribute to the development of new intervention approaches and health policies. From this point of view, this study was planned to test the predictive role of HL on HPPB among Turkish health workers who act as a bridge between the society and the health system.

The following questions were sought to be answered in the study:

- What are the levels of HL and HPPB among Turkish nurses and allied health workers?
- Is there a significant relationship between HL and HPPB in Turkish nurses and allied health workers?
- Do the HL levels of healthcare workers predict HPPB?
- Is there a significant difference between the descriptive characteristics of Turkish nurses and allied health workers and HL and HPPB?

## 2 Research Methodology

### 2.1 Study Design and Participants

This study was designed as a descriptive and cross‐sectional study. The snowball sampling method was used in the sample selection of the study. The sample group consists of Turkish nurses and allied health workers (doctors, midwives, physiotherapists, dietitians, psychologists and others) who are actively working in hospitals in Türkiye, have at least 6 months of work experience in their profession, participate in the study voluntarily, and fill out the data collection form completely.

In the literature; in the post hoc power analysis; a power of over 0.8 indicates that the sample size is sufficient (Cohen 1988; Polit and Beck 2017). To determine the adequacy of the sample size, a post hoc power analysis was conducted based on the correlation coefficient (*r* = 0.466), with a significance level set at *α* = 0.05. A post hoc power analysis was conducted using G*Power (version 3.1.9.2), and with a total sample size of 403 participants, the statistical power (1—β) of the study was calculated to be approximately 1.00. This result indicates that the study had sufficient power to detect a medium‐to‐large effect size with a high level of confidence.

Inclusion and Exclusion Criteria:

- Individuals who met the following criteria were included in the study:
- Actively working in hospitals or healthcare institutions in Turkey,
- Agreeing to participate voluntarily in the study,
- Completing the data collection form completely.

Those on administrative or medical leave during the data collection period and those who provided incomplete or inconsistent data were excluded from the study.

### 2.2 Instruments

Data were collected using “Introductory Information Form”, “Health Literacy Scale Turkish Version”, and “Promotive and Protective Health Behaviors Scale”.

#### 2.2.1 Introductory Information Form

The form was prepared by the researchers after the review of the relevant literature (Zeng et al. 2021; Yogurtcu and Ozturk Haney 2022). The form consisted of nine questions asking about the participants' information about their profession, age, gender, marital status, educational level, the hospital they work for, the unit they work for, working hours, and whether they practice their profession with passion.

#### 2.2.2 Health Literacy Scale—Turkish Version (HLS‐14)

The scale was developed by Suka et al. (2015) to measure the HL level of individuals. Turkoglu and Kilic (2021) conducted the Turkish validity and reliability study of the scale. HLS consists of three factors: functional health literacy (5 items), interactive health literacy (5 items), and critical health literacy (4 items), and a total of 14 items. Likert‐type 5‐point scale, ranging from “1 = strongly disagree” to “5 = strongly agree”. The minimum score obtained from the scale is 14, the maximum score is 70. The higher the score, the higher the level of HL. The Cronbach alpha reliability coefficient reported in the validity‐reliability study of the scale is 0.85 (Turkoglu and Kilic 2021). The Cronbach Alpha reliability coefficient of the scale in this study is 0.84.

#### 2.2.3 Promotive and Protective Health Behaviours Scale (HPPBS)

The scale developed by Bostan et al. (2016) to measure the HPPB levels of individuals consists of three subscales (physical, psychosocial and protection) and a total of 24 items. The scale is a five‐point Likert type. Scale items are scored between 1 and 5 points (1 = never, 2 = very rarely, 3 = sometimes, 4 = most of the time, 5 = always). There are reverse items in the scale (items 1,3,4,5,12,13,14,23) The lowest score that can be obtained from HPPB is 24, and the highest score is 120. An increase in the score obtained from the scale indicates better HPPB. The Cronbach alpha reliability coefficient reported in the validity‐reliability study of the scale is 0.83 (Bostan et al. 2016). The Cronbach Alpha reliability coefficient of the scale in this study was calculated as 0.82.

### 2.3 Data Collection

After obtaining the necessary permissions for the study, data collection was carried out between January and May 2024. Data collection was carried out via an online survey. The researchers sent the form to all healthcare workers they could reach online via social media and asked them to fill it out. Each participant was allowed to answer the online survey only once. It took approximately 7–10 min to complete the data collection tools.

### 2.4 Data Analysis

JASP 0.18.3.0, Jamovi 2.3.21, and SPSS 26 programs were used for data analysis. The Descriptive statistical methods (number, percentage, min‐max values, mean, and standard deviation) were used to evaluate the data. Whether the data used was distributed normally or not was tested with the Kurtosis‐Skewness values. The Student's t and One Way ANOVA tests were applied. The Bonferroni test was performed for post hoc analyses. Additionally, simple regression were used to determine the effect. While evaluating the results obtained, 95% confidence interval and *p* < 0.05 error level were taken into consideration.

## 3 Results

### 3.1 Demographic Characteristics

The average age of the healthcare workers participating in the study was 32.83 ± 8.14 and the mean professional experience was 8.7 ± 7.32. Forty percent were nurses, 54.6% had a bachelor's degree, and 46.7% had income level equal to the expenses. The rate of those who practise their profession with passion was found to be 72.7%, the rate of those who know the concept of health literacy was found to be 57.1%, the rate of those who did not consume alcohol was found to be 55.1%, and the rate of those who smoked cigarettes was found to be 37.5%. Moreover, it was found that 52.1% of the participants did not do sports and 81.1% did not have a chronic disease. Table 1 shows the demographic characteristics of the Turkish nurses and allied health workers.

**TABLE 1:** Descriptive characteristics of participants.

Variables		*n*	%
Age	19–35	281	69.7
	36–52	110	27.3
	53 and over	12	3.0
Profession	Physician	54	13.4
	Nurse	161	40.0
	Physiotherapist	23	5.7
	Dietitian	16	4.0
	Pharmacist	43	10.7
	Other	106	26.3
Professional years	0–13	330	81.9
	14–27	60	14.9
	27 and above	13	3.2
Educational level	High school	29	7.2
	Bachelor's degree	220	54.6
	Master's degree	117	29.0
	Doctor's degree	37	9.2
Income level	Income equals expenses	188	46.7
	Income is more than expenses	98	24.3
	Income is less than expenses	117	29.0
Practicing one's profession with passion	Yes	293	72.7
	No	110	27.3
Knowing the concept of health literacy	Yes	230	57.1
	No	173	42.9
Alcohol consumption	Yes	181	44.9
	No	222	55.1
Cigarette consumption	Yes	151	37.5
	No	252	62.5
Doing sports	Yes	193	47.9
	No	210	52.1
Presence of a chronic disease	Yes	76	18.9
	No	327	81.1
Age	Mean ± SD 32.83 ± 8.14		
Professional years	Mean ± SD 8.7 ± 7.32		
Total		403	100

### 3.2 The Relationship Between the Health Literacy and Health‐Promoting and Protective Behaviours of the Turkish Nurses and Allied Health Workers

The total score of HPPBS was found to be 82.32 ± 10.68 and its sub‐dimensions were Physical (32.66 ± 5.00), Psychosocial (20.06 ± 3.42) and Protection (29.59 ± 4.81), respectively. The total score of HLS was found to be 52.17 ± 17.76 and its sub‐dimensions were Functional HL (17.76 ± 3.93), Interactive HL (18.81 ± 3.10) and Critical HL (15.59 ± 2.78), respectively. It was determined that the average HLS and HPPBS score of participants was at a moderate level (Table 2).

**TABLE 2:** Correlation analysis results of HLS, HPPBS and its sub‐scales and scores.

Scales and sub‐scales	Promotive and protective health behaviours (82.32 ± 10.68)	Physical (32.66 ± 5.00)	Psychosocial (20.06 ± 3.42)	Protection (29.59 ± 4.81)
Health literacy (52.17 ± 7.76)	*r* = 0.460 *p* = 0.000*	*r* = 0.343 *p* = 0.000*	*r* = 0.361 *p* = 0.000*	*r* = 0.407 *p* = 0.000*
Functional health literacy (17.76 ± 3.93)	*r* = 0.281 *p* = 0.000*	*r* = 0.233 *p* = 0.000*	*r* = 0.218 *p* = 0.000*	*r* = 0.227 *p* = 0.000*
Interactive health literacy (18.81 ± 3.10)	*r* = 0.410 *p* = 0.000*	*r* = 0.297 *p* = 0.000*	*r* = 0.322 *p* = 0.000*	*r* = 0.374 *p* = 0.000*
Critical health literacy (15.59 ± 2.78)	*r* = 0.426 *p* = 0.000*	*r* = 0.296 *p* = 0.000*	*r* = 0.339 *p* = 0.000*	*r* = 0.396 *p* = 0.000*

* *p* < 0.01.

**FIGURE 1:**
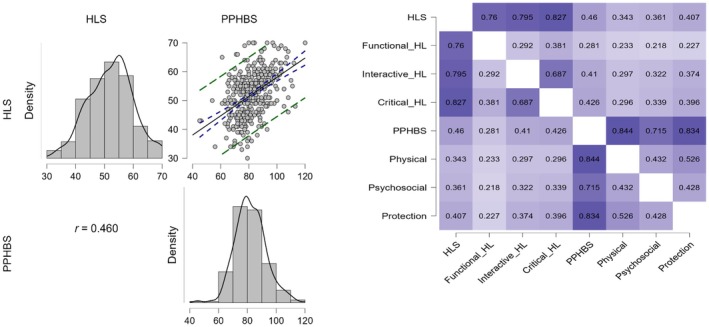
Relationship, density, and scatter plot between the scales and subscales (correlation analysis results between HLS and HPPBS).

According to the results of the Pearson correlation analysis, a positive, moderately statistically significant relationship was found between HLS and HPPBS (*r* = 0.460, *p* = 0.000) (Table 2). A positive, statistically significant difference relationship was found between HLS and PPHBS sub‐scales; the Physical sub‐scale (*r* = 0.343, *p* = 0.000), the Psychosocial sub‐scale (*r* = 0.361, *p* = 0.000), and the Protection sub‐scale (*r* = 0.407, *p* = 0.000), respectively. Moreover, A positive, statistically significant difference relationship was found between HPPBS and HLS sub‐scales; the Functional HL sub‐scale (*r* = 0.281, *p* = 0.000), the Interactive HL sub‐scale (*r* = 0.410, *p* = 0.000), and the Critical HL sub‐scale (*r* = 0.426, p = 0.000), respectively (Table 2). The relationship between scales, density and distribution graph is as shown in the figure (Figure 1 and Table 2).

### 3.3 Regression Analysis Results for Health Literacy's Prediction of Health‐Promoting and Protective Behaviours

**FIGURE 2:**
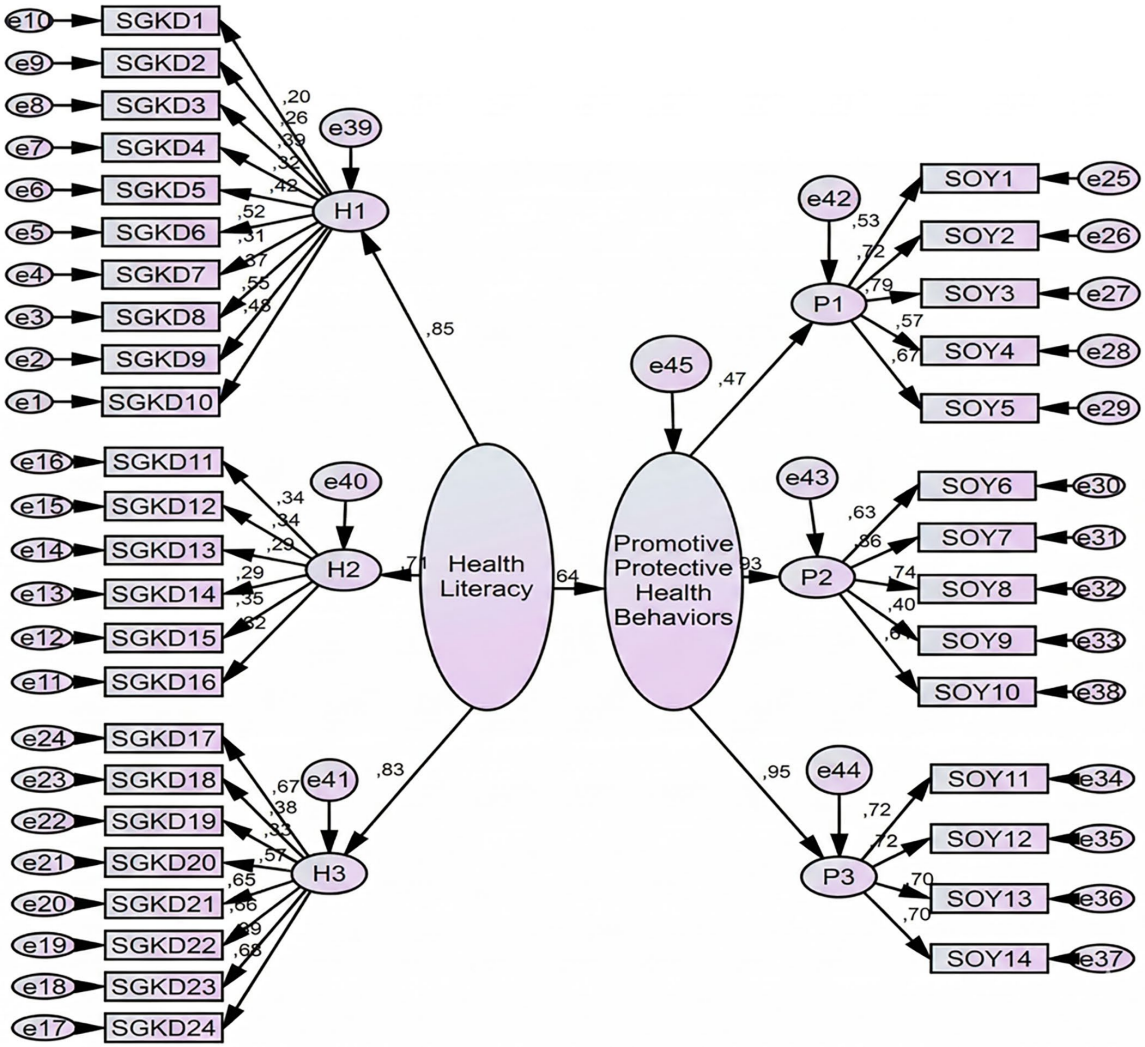
The effect of life satisfaction on happiness with AMOS analysis results.

Simple regression analysis was conducted to investigate the effect of HL on HPPB (Model 1). HL explained 21.1% of the change in HPPB (*R* ^2^ = 0.211). A positive and significant relationship was observed between HL and HPPB (β = 0.460, *p* < 0.05). A one‐unit increase in HL caused an increase of 0.64 in HPPB (Table 3). In addition, Amos analysis confirmed that HL has a positive effect on HPPB (Figure 2), and the goodness of adjustment values were found to be at the desired level (Table 4).

**TABLE 3:** Simple regression and multiple analysis results for health literacy's prediction of health‐promoting and protective behaviours.

Independent variable	Dependant variable	Beta	*t*	*p*	*ß*	F	Model (*p*)	*R* ^2^
Model 1	Constant	49.307	15.309	0.000		107.418	0.000*	0.211
Health Literacy	Promotive protective Health behaviours	0.633	10.364	0.000	0.460		0.000*	
Model 2	Constant	24.863	9.221	0.000		37.130	0.000*	0.218
Physical	Promotive protective Health behaviours	0.19	2.260	0.024	0.122			
Psychosocial		0.45	3.900	0.000	0.198			
Protection		0.41	4.772	0.000	0.257			
Model 3	Constant	48.937	14.873	0.000		38.059	0.000*	0.222
Functional HLS	Health literacy	0.358	2.757	0.006	0.132			
Interactice HLS		0.741	3.541	0.000	0.215			
Critical HLS		0.874	3.624	0.000	0.228			

* *p* < 0.05.

**TABLE 4:** The effect of health literacy on health‐promoting and protective behaviours with amos analysis results.

Impact	Estimate	Standard error	*t*	*p*	Result
Health literacy ➔ Promotive and protective health behaviours	0.642	0.110	4.830	0.03*	Acceptable
Adjusment values: CMIN/DF: 2.199, RMSEA: 0.050, GFI: 0.831, AGFI: 0.810, SRMR: 0.06					

* *p* < 0.05.

Structural equation modelling was conducted using the AMOS program to determine the impact of health literacy on health‐promoting and protective behaviours. The analysis revealed that health literacy had a positive and significant effect on health‐promoting and protective behaviours (*β* = 0.642, *t* = 4.830, *p* < 0.001). This finding indicates that as health literacy levels increase, individuals' health‐promoting and protective behaviours also increase. Analysis of the model's fit indices revealed an acceptable level of fit (CMIN/DF = 2.199, RMSEA = 0.050, GFI = 0.831, AGFI = 0.810, SRMR = 0.06). These values indicate that the model fits the data well. Therefore, the results demonstrate that health literacy is a significant predictor of health behaviours and that this variable significantly influences individuals' health‐promoting and protective tendencies (Table 4).

Additionally, the effects between the sub‐dimensions of the structure and its dimensions were tested with simple regression analysis (Model 2). According to the results of this analysis, the effects of independent variables on HPPB were examined. Variables such as Physical (*ß* = 0.19, *p* = 0.024), Psychosocial (*ß* = 0.45, *p* = 0.000), and Protection (*ß* = 0.41, *p* = 0.000) have a significant effect on health deterioration. Psychosocial and Protection sub‐dimensions have the strongest people in terms of effect sizes, while Physical shows a stronger effect. The overall significance of the model is evident with *F* = 37.130 and *p* = 0.000, and the total explanation rate of the independent variables on HPPB is stated as *R* ^2^ = 0.218. This shows that the model explains 21.8% of the dependent variable (Table 3).

In the Model, the effect of independent variables such as Functional HLS, Interactive HLS and Critical HLS on HL was evaluated. Critical HLS (*ß* = 0.874, *p* = 0.000) showed the strongest effect, while Interactive HLS (*ß* = 0.741, *p* = 0.000) and Functional HLS (*ß* = 0.358, *p* = 0.006) also had a significant effect. The overall significance of the model is *F* = 38.059 and *p* = 0.000, with a high structure; the total explanation rate of independent variables on HL changes to *R* ^2^ = 0.222. This shows that the effect of independent variables on HL is 22.2% (Table 3).

### 3.4 Comparison of Scale Scores of Turkish Nurses and Allied Health Workers With Their Descriptive Characteristics

When the mean HLS scores were examined, while a statistically significant difference was found between HLS and age, profession, educational level, practicing one's profession with passion, knowing the concept of HL, alcohol consumption, and doing sports (*p* < 0.05), no statistically significant difference was found between professional years, income level, cigarette consumption, and presence of a chronic disease and HLS (*p* > 0.05). As a result of the post hoc (Bonferroni) analyses, the mean HLS score of those aged 19–35 was found higher than that of those aged 36–52 and 53 and above and the mean HLS score of physicians was found higher than that of pharmacists and physiotherapists, and that of nurses was found higher than that of physiotherapists. When the educational level was examined, the mean HLS score of those who had a bachelor's degree and master's degree was found higher than that of high school graduates; the mean HLS score of those who had Doctor's degree was found to be higher than that of high school and undergraduate graduates. Additionally, the mean HLS score of those who practiced their profession with passion was found higher than that of those who did not, those who knew the concept of health literacy than those who did not, those who did not consume alcohol than those who did, and those who did sports than those who did not (Table 5).

**TABLE 5:** Comparison of scores of HLS and HPPBS according to descriptive characteristics.

Variables		HLS			PPHBS		
		X̄ ± SD	*t*/*F*	*p*/Bnf	X̄ ± SD	*t*/*F*	*p*/Bnf
Age^b^	19–35	53.17 ± 7.46	10.983	0.000*	82.20 ± 11.07	1.125	0.326
	36–52	50.40 ± 7.85			83.07 ± 10.09		
	53 and over	44.91 ± 7.76			78.33 ± 5.91		
Profession^b^	Physician	55.25 ± 7.15	4.720	0.001*	85.38 ± 10.18	4.697	0.000*
	Nurse	52.81 ± 7.36			82.55 ± 10.07		
	Physiotherapist	47.78 ± 9.10			81.65 ± 10.08		
	Dietitian	50.56 ± 7.08			91.06 ± 11.59		
	Pharmacist	50.48 ± 9.97			81.44 ± 11.95		
	Other	51.50 ± 6.80			79.60 ± 10.41		
Professional years^b^	0–13	52.78 ± 7.55	1.189	0.219	82.48 ± 11.12	0.881	0.666
	14–27	50.10 ± 8.21			82.46 ± 8.35		
	27 and above	46.15 ± 7.13			77.61 ± 8.32		
Educational level^b^	High school	47.58 ± 6.77	8.605	0.000*	74.68 ± 10.13	11.479	0.000*
	Bachelor's degree	51.52 ± 7.04			81.45 ± 9.84		
	Master's degree	53.20 ± 8.15			83.82 ± 10.54		
	Doctor's degree	56.35 ± 8.96			88.78 ± 12.11		
Income level^b^	Income equals expenses	51.90 ± 7.63	0.566	0.568	82.40 ± 10.56	0.905	0.406
	Income is more than expenses	52.89 ± 8.75			83.31 ± 11.65		
	Income is less than expenses	51.99 ± 7.08			81.35 ± 10.03		
Practicing one's profession with passion	Yes	52.82 ± 7.52	2.775	0.006*	83.40 ± 10.58	3.345	0.010*
	No	50.43 ± 8.15			79.45 ± 10.48		
Knowing the concept of health literacy^a^	Yes	54.51 ± 7.36	7.794	0.000*	83.95 ± 11.22	3.584	0.000*
	No	48.93 ± 7.07			80.15 ± 9.54		
Alcohol consumption^a^	Yes	50.95 ± 8.20	−2.882	0.005*	81.79 ± 9.98	−0.588	0.557
	No	53.17 ± 7.25			82.60 ± 11.24		
Cigarette consumption^a^	Yes	51.35 ± 8.09	−1.637	0.102	79.47 ± 10.36	−0.4.237	0.000*
	No	52.66 ± 7.53			84.03 ± 10.53		
Doing sports^a^	Yes	53.07 ± 7.58	2.239	0.026*	85.10 ± 10.94	5.170	0.000*
	No	51.34 ± 7.84			79.67 ± 9.80		
Presence of a chronic disease^a^	Yes	51.23 ± 8.50	−1.168	0.243	80.27 ± 9.21	−1.861	0.040*
	No	52.39 ± 7.57			82.80 ± 10.96		

Abbreviations: Bnf, Bonferroni test; HLS, health literacy scale; HPPBS, promotive and protective health behaviours scale.

* *p* < 0.05.

^a^ *t*, Student's *t*‐test.

^b^ *F*, one way ANOVA.

When the mean HPPBS scores were examined, while a statistically significant difference was found between HPPBS and profession, educational level, practicing one's profession with passion, knowing the concept of HL, cigarette consumption, doing sports, and presence of a chronic disease (*p* < 0.05); no statistically significant difference was found between age, professional years, income level, and alcohol consumption (*p* > 0.05). The Post hoc (Bonferroni) analysis showed that the mean HPPBS scores of dietitians were higher than that of physicians and nurses; the mean HPPBS scores of physicians and dietitians were higher than that of other professional groups. Moreover, it was determined that the mean HPPBS scores of undergraduate, MSc, and PhD graduates were higher than those of high school graduates. Furthermore, the mean HPPBS score of those who practiced their profession with passion was found higher than that of those who did not, those who knew the concept of health literacy than those who did not, those who did not consume cigarette than those who did, and those who did sports than those who did not, and those who had a chronic disease than who did not (Table 5).

## 4 Discussion

In this study examining the predictive power of the HL level of Turkish nurses and allied health workers on HPPB, the HL level of the participants was found to be moderate (52.17 ± 7.76). This is similar to previous studies (Bukecik and Adana 2021; Koca and Deniz 2024). This shows that the desired HL level has not yet been achieved. Morever, it is reported in the literature that low HL levels are associated with poor health outcomes, ineffective and inadequate use of health services (Berkman et al. 2011; Allen‐Meares et al. 2020) It is important for healthcare workers who provide health services to the community to have a high HL level in terms of creating awareness about HL in the community, increasing HL levels and producing positive health outcomes (Durmaz et al. 2016). Therefore, there is a need to develop interventions and strategies that will increase the HL levels of healthcare workers.

In this study, it was determined that the participants had moderate HPPB (82.32 ± 10.68). When other studies in the literature are examined, it is seen that the studies are similar (Yanik and Nogay 2017; Zeng et al. 2021; Yogurtcu and Ozturk Haney 2022). Healthcare workers have important duties in introducing healthy lifestyle behaviours to the society and in protecting and improving health. It is important for healthcare workers, who largely affect the group they serve due to their professional responsibilities and should be role models for the society, to adopt health‐promoting and protective behaviours and adapt them to their own lives (Soykan and Sengul 2021). Therefore, it may be useful to develop interventions that will improve the HPPB levels of healthcare workers.

Our correlation analysis results showed that there is a positive, moderately significant relationship between HLS and HPPBS. Other studies in the literature also reported a relationship between HL and HPPB (Kazak et al. 2021; Soykan and Sengul 2021; Zeng et al. 2021; Yogurtcu and Ozturk Haney 2022). In this respect, our findings are similar to the literature. Our regression analysis results showed that HL level explained 21.1% of the change in HPPB and was a significant predictor. The findings of this study are very important since no other study has been found investigating the predictive effect of HL level on HPPB in nurses and allied health workers. Additionally, this result suggests that HL levels should be considered an important part of HPPB studies for healthcare workers. Moreover, it is anticipated that research results examining the predictive power of HL on HPPB in different groups and societies will contribute to a more in‐depth understanding of the effects of HL and HPPB.

Although HL explained 21.1% of the variance in HPPB in our sample, it is clear that health‐promoting behaviours are not solely determined by HL. These behaviours may be influenced by a broader range of factors, including sociodemographic characteristics (age, education, income), work‐related conditions (shift schedule, workload, occupational group, organization's health culture), individual psychosocial factors (self‐efficacy, health beliefs, intention, perceived barriers), and digital competencies (e‐health literacy, numerical/numerical literacy). Therefore, in future studies with health workers, testing multivariate models that incorporate these variables under the same umbrella would be appropriate. Testing the direct, indirect, or conditional (interactive) effects of HL on HPPB, preferably using hierarchical regression, structural equation modelling, or multilevel approaches, would increase the explained variance and reveal the relationship in a more nuanced manner.

The study findings showed that there was a statistically significant difference between the HL level and sociodemographic variables (age, profession, education level, passionately practicing the profession, knowing the concept of HL, alcohol consumption and doing sports). In the study, HL scores decreased as the age group of the participants increased. While some studies reported no significant difference between age and HL level (Kazak et al. 2021); some studies reported a significant difference between age and HL level (Koca and Deniz 2024). The reason for this may be that HL has become an increasingly popular concept in recent years, and it has been included in the educational curriculum in recent years. Furthermore, some studies in the literature highlight the increasing digitalization of health information as a key reason why older generations exhibit lower health literacy. Qiu et al. (2025) reported that, using ages 45–54 as a reference, the 55+ age group had significantly lower digital health literacy scores, while the 25–34 age group had higher scores. This suggests that older individuals face disadvantages in accessing health information due to their lack of digital literacy. Another cohort study examining the relationship between health literacy, education, and health outcomes (Yamashita and Brown 2017) reported that the “age” variable lost statistical significance. This study emphasized that the differences observed with age are not due to age itself, but to the different educational experiences of the respective generation. Accordingly, a more in‐depth examination of age and HL level and an explanation of possible variables may be more useful.

In addition, the group with the highest HL levels was physicians. This finding is parallel to other study findings in the literature (Deniz et al. 2018; Alipour and Payandeh 2022). This may be related to the education level of physicians. It is thought that initiatives aimed at increasing the HL level of other healthcare workers would be beneficial.

In the study, it was determined that HL levels increased as the level of education increased. There are many studies in the literature showing that HL scores increase as the level of education increases (Bukecik and Adana 2021; Koca and Deniz 2024; Sørensen et al. 2015). Our study findings support the literature.

In our study, it was determined that those who knew the concept of HL, passionately practicing their profession, did not consume alcohol and did sports had better HL levels. In previous studies, it was reported that those who knew HL and did sports had better HL (Bukecik and Adana 2021). These findings are also in line with previous literature that found a positive association between HL awareness and participation in physical activity with higher HL levels. This correlation supports the validity of our results and demonstrates the importance of behavioural and motivational parameters in shaping health literacy. In this respect, our findings confirm the literature.

In our study, it was determined that those who knew the concept of HL, passionately practicing their profession, did not smoke, did sports and did not have chronic diseases had better HPPB levels. Previous studies reported that those who did sports and did not have chronic diseases had better health behaviours (Yanik and Nogay 2017; Yildiz et al. 2022). Previous studies are supported by literature emphasizing that individuals who engage in physical activity and those who do not have chronic health problems are more likely to exhibit healthier attitudes and behaviours (Yanik and Nogay 2017; Yildiz et al. 2022). This study clearly shows that individual lifestyle and professional motivation are important parameters supporting HPPB of healthcare workers. In this respect, our findings were consistent with the literature.

As the level of education increased, HPPBS scores increased. Other studies in the literature have reported that health‐promoting and protective behaviours increase as the level of education increases (Kazak et al. 2021; Zeng et al. 2021). In this respect, the findings are similar to the literature.

### 4.1 Limitations

Although our study has strengths as the first study examining the predictive power of HL of Turkish nurses and allied health workers in Türkiye on HPPB, it has some limitations. The biggest limitation of the study is the number of samples. Therefore, it cannot be generalized to all healthcare workers. Since the study data were obtained through a cross‐sectional design and an online self‐report survey, participants' responses are susceptible to social desirability bias.

## 5 Conclusion

This study showed that health literacy and health‐promoting and protective behaviours of Turkish nurses and allied health workers were at a moderate level. The results showed that there was a significant relationship between HL levels and HPPB, and that HPPB increased as HL increased. Regression analysis results showed that HL explained 21.1% of the change in HPPB and was an important predictor of HPPB. While a statistically significant difference was found between HLS and age, occupation, level of education, passionately practicing one's profession, knowing the concept of HL, alcohol consumption, smoking, and doing sports, a statistically significant difference was found between HPPBS and occupation, level of education, passionately practicing one's profession, knowing the concept of HL, smoking, alcohol consumption, doing sports, and the presence of a chronic disease. These findings highlight the need for evidence‐based intervention programs to increase HL among Turkish nurses and allied health workers, not only to improve their own health but also to support the health of the individuals they care for. Adding health literacy‐based content to continuing professional development programs, supporting workplace health promotion initiatives, and developing targeted health communication strategies may be effective approaches. Health policymakers should establish supportive environments and structures to sustain healthcare workers' HPPB; this can strengthen a culture of prevention, improve patient outcomes, and reduce health disparities.

## 6 Practice Implications

The findings of this study offer significant contributions to the field of health promotion by highlighting the critical role of HL in shaping HPPB among Turkish nurses and allied health workers. These results underscore the need for structured and sustainable intervention strategies aimed at enhancing HL competencies within healthcare settings. This study, on the one hand, develops theories about HPPB, and on the other hand, provides a reference value for intervention programs. Such initiatives can lead to improved self‐care practices, better patient education, and overall enhancement of community health outcomes. The study results are important for both researchers and health policy makers. It is important to improve HL levels in order to increase HPPB of health workers. Based on the findings of this study, there is a need for interventions and training programs designed to improve HL and HPPB of Turkish nurses and allied health workers. Ultimately, advancing health literacy among healthcare workers is not only a professional development priority but also a strategic investment in the quality and effectiveness of healthcare systems.

## Author Contributions

D.D.A.: Conceptualization, writing – original draft, investigation, methodology, validation, writing – review and editing, formal analysis, project administration, data curation, supervision. A.Y.: Conceptualization; investigation; writing – original draft; methodology; validation; project administration; data curation.

## Funding

This work was supported by Türkiye Bilimsel ve Teknolojik Araştırma Kurumu.

## Disclosure

Wiley and TÜBİTAK ULAKBİM have an agreement. https://authorservices.wiley.com/author‐resources/Journal‐Authors/open‐access/affiliation‐policies‐payments/tubitak‐agreement.html.

## Ethics Statement

At the outset, written approvals were obtained from Munzur University Non‐Invasive Ethics Committee (Date:30.11.2023, No:2023/13–17) to conduct the study. The study was conducted in accordance with the Declaration of Helsinki. Confidentiality, privacy, and the well‐being and human rights of the participants were protected throughout the study. Participation in the study was voluntary, and the participants were informed that they were free to withdraw from the study at any time.

## Conflicts of Interest

The authors declare no conflicts of interest.

## Data Availability

The data that support the findings of this study are available from the corresponding author upon reasonable request.
